# Association of Dental Colleges

**Published:** 1867-05

**Authors:** 


*Association of Dental Colleges.*—We wish to call the attention of our readers especially to the proceedings of the Association of Dental Colleges—in our judgement the most important subject in Dentistry that can engage the mind of the reader or employ the pen of the journalist. We confess to a feeling of disappointment that the Editors of so widely circulated a journal as the *Cosmos*, should have passed over, without notice certain minutes in those proceedings which record an action that must have a decided influence upon Dental Education.

That one of the Colleges represented in that Association—and one of the most influential—should have refused to co-operate with the others in their effort to improve the system of education; still further that it should have determined to pursue a course which the others regard injurious to the cause of Collegiate Education—is a matter of gravest importance. It challenges the censure or approval of the Profession, and is a subject which must necessarily be discussed. Our pages will be open to any communications, on either side of the question, which shall be written with fairness and courtesy. As Editors we cannot permit any exclusion from our journal of temperate discussion of a subject so important as Dental Education. But as friends of education, and Professors in a Dental College, we think it our duty to record our disapproval of the *Secession* of the Pennsylvania College.

